# Supplementary figures and images for: IntegrAlign: a comprehensive tool for multi-immunofluorescence panel integration through image alignment
Source: Bioinformatics. 2025 Oct 16;41(11):btaf567. doi: 10.1093/bioinformatics/btaf567 (PMC12582367; doi:10.1093/bioinformatics/btaf567)

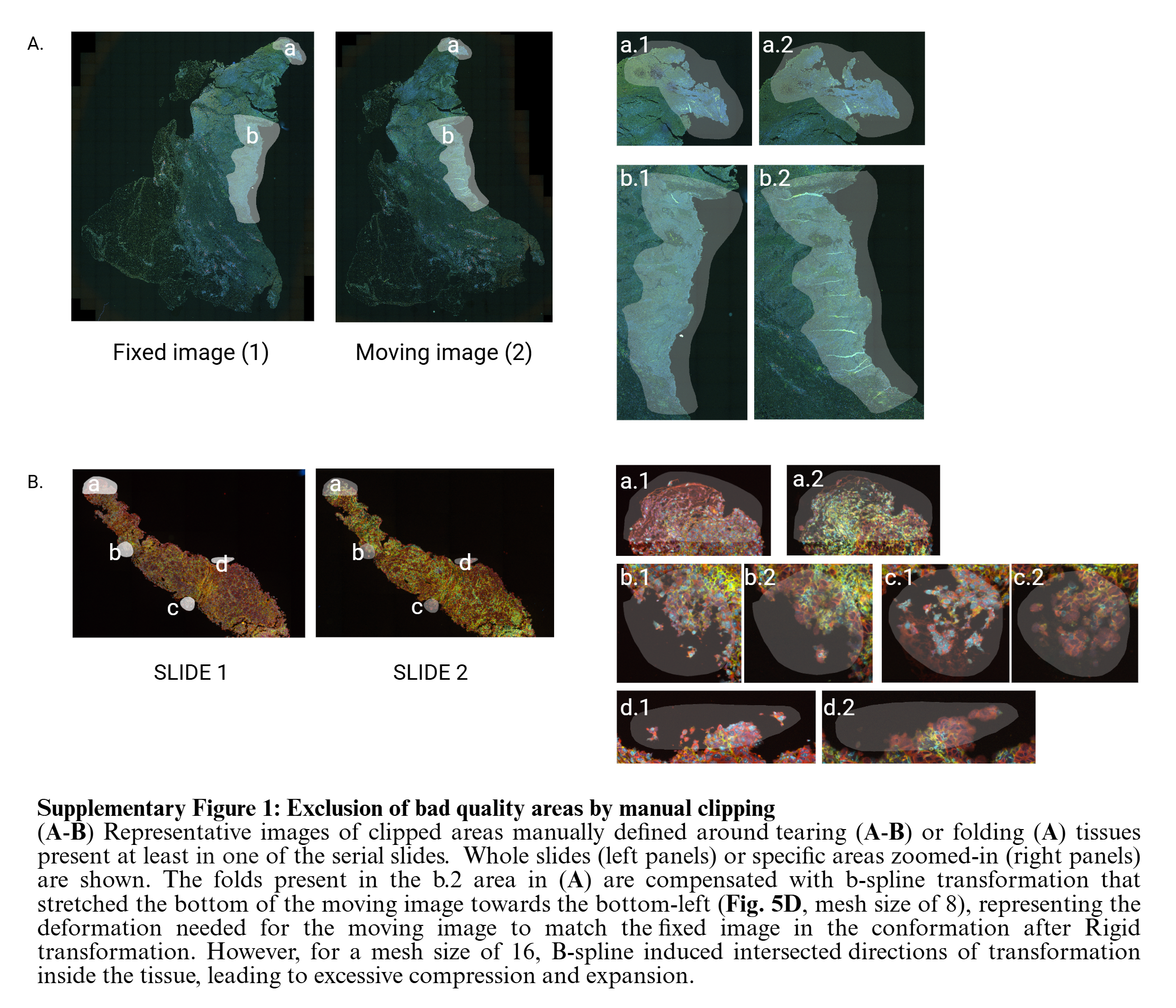

Supplement: btaf567_Supplementary_Data [file btaf567_supplementary_data.zip › supp-fig1_IntegrAlign.png]

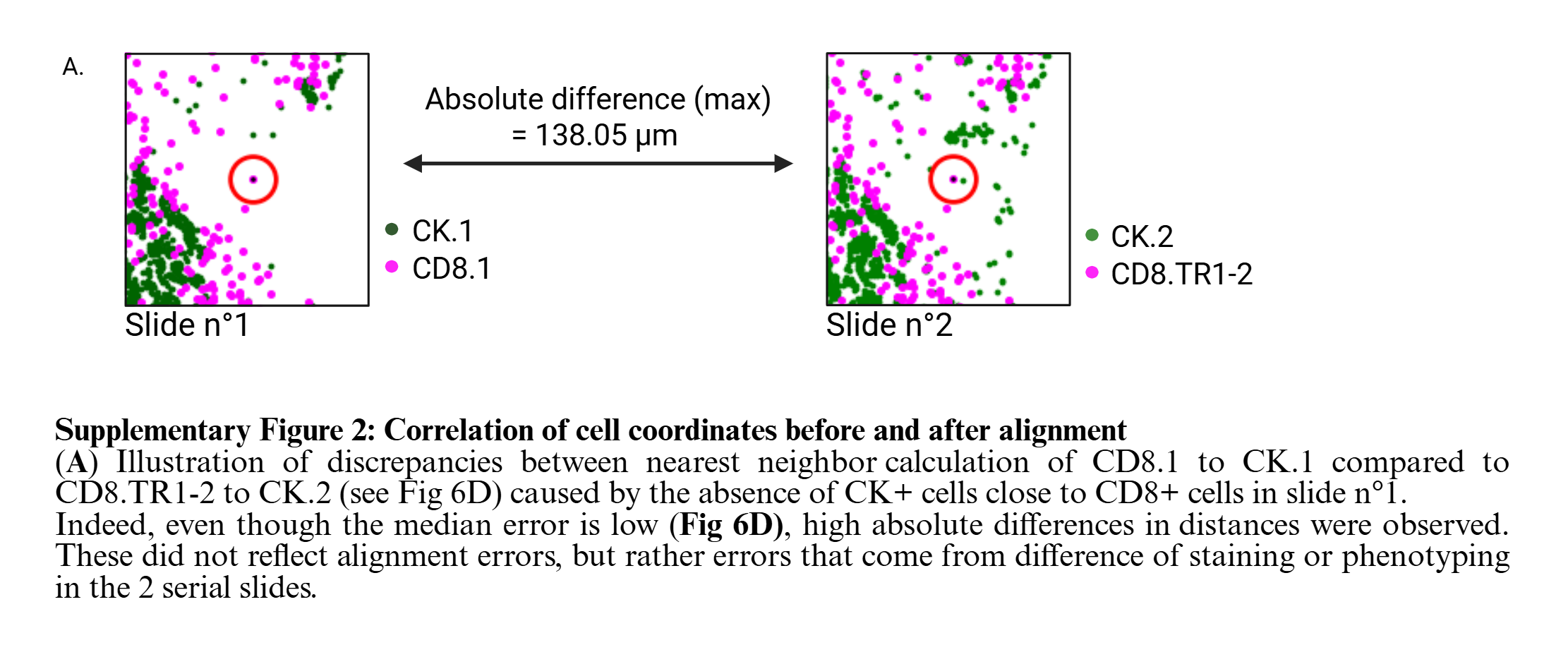

Supplement: btaf567_Supplementary_Data [file btaf567_supplementary_data.zip › supp-fig2_IntegrAlign.png]

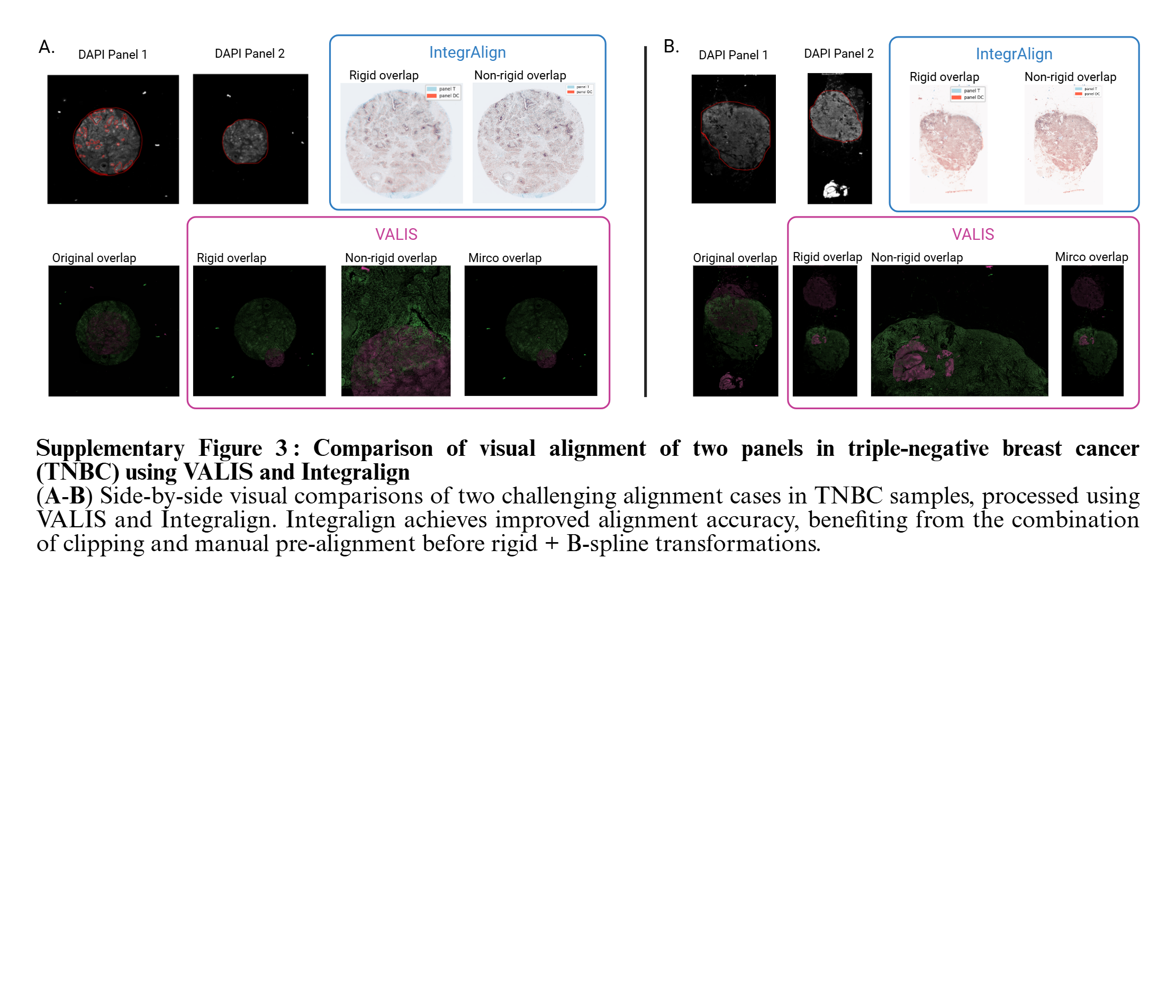

Supplement: btaf567_Supplementary_Data [file btaf567_supplementary_data.zip › supp-fig3_IntegrAlign.png]

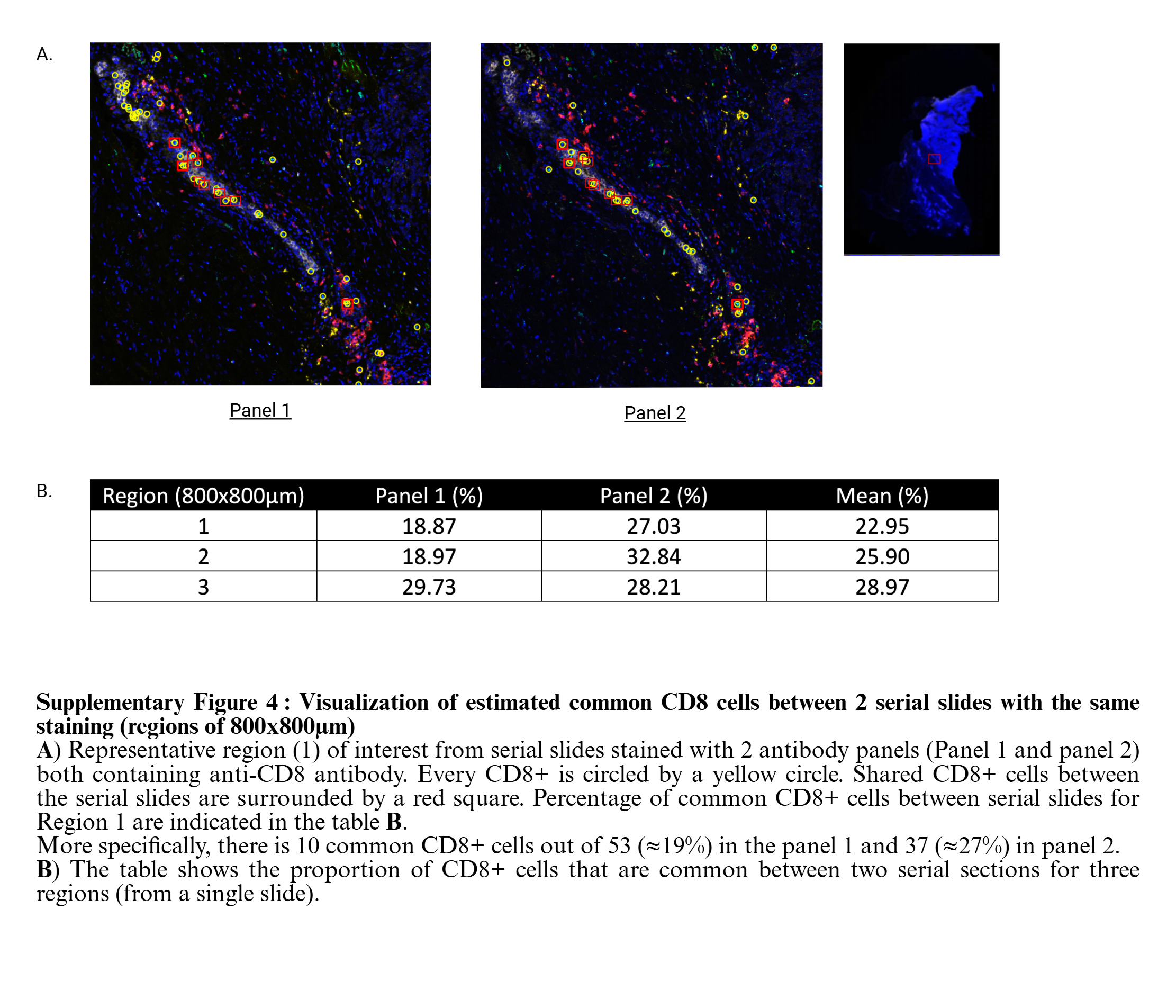

Supplement: btaf567_Supplementary_Data [file btaf567_supplementary_data.zip › supp-fig4_IntegrAlign.png]
